# Hysterosalpingography Findings and Jimah Ratio of the Uterine Cavity in Women with Infertility in Central Region, Ghana

**DOI:** 10.1155/2020/6697653

**Published:** 2020-12-14

**Authors:** Bashiru Babatunde Jimah, Philip Gorleku, Anthony Baffour Appiah

**Affiliations:** ^1^Department of Medical Imaging, School of Medical Sciences, University of Cape Coast, Cape Coast, Ghana; ^2^Ghana Field Epidemiology and Laboratory Training Programme, University of Ghana, Accra, Ghana

## Abstract

**Background:**

Infertility affects from 1.3% to 25.7% of couples worldwide and, especially, from 14.5% to 16.4% in Africa. Hysterosalpingography (HSG) is a diagnostic modality that is considered both common and efficient. It is used to investigate abnormalities of the uterine cavity and fallopian tubes. This study assessed the spectrum of findings on HSG among women with infertility in the Central Region (Ghana).

**Methods:**

We conducted a prospective cross-sectional study to examine 203 infertile women undergoing HSG work-up at the Cape Coast Teaching Hospital. The exclusion criteria were acute infection of the vagina or cervix and active vaginal bleeding or pregnancy. Data were entered with Microsoft Excel and analyzed using SPSS version 21.

**Results:**

A total of 203 women were enrolled, and eighty-five (41.87%) of the women had at least one or more abnormalities. The mean age was 32.9 years with majority of the women within 30–39 years (61.08%). More than half (50.74%) of the women presented with secondary infertility, while age of women (*p*=0.004) and duration of infertility (0.034) were found to be in association with the type of infertility. Uterine findings were predominantly capacious uterine cavity (45.1%) and uterine fibroids (33.3%), while fallopian tube findings included bilateral blockage (24.2%), right unilateral proximal blockage (17.7%), loculated spillage (16.1%), and left unilateral proximal blockage (16.1%). The range of normal uterine cavity size, measured as ratio (*Jimah ratio*) of intercornual diameter to interiliac diameter was 0.2–0.45, with a mean of 0.36.

**Conclusion:**

Secondary infertility was the commonest indication for HSG in the study, and a significant proportion of infertile women had abnormalities. Abnormalities were higher in the fallopian tubes than the uterus, while capacious uterine cavity, uterine fibroid, and bilaterally blocked tubes were the top three abnormalities found.

## 1. Introduction

Infertility is a disease of the reproductive system defined by the failure to achieve a clinical pregnancy after 12 months or more of regular unprotected sexual intercourse [1, 2]. Generally, infertility can be primary or secondary. Primary infertility is defined as “the absence of pregnancy for a couple who desire a child after 12 months or more of regular unprotected sexual intercourse, during which they have not used any contraceptives [3]. Secondary infertility is defined as “the absence of a live birth for women who desire a child and have been in a union for at least five years since their last live birth, during which they did not use any contraceptives [3].

Infertility is a gynecological disorder with associated medical, psychological, and economic problems, and it affects 1.3% to 25.7% of couples worldwide [4, 5]. Infertility prevalence is highest in South Asia, sub-Saharan Africa, North Africa/Middle East, and Central/Eastern Europe and Central Asia [3]. Generally, the prevalence of secondary infertility is higher than primary infertility [3]. In sub-Saharan Africa, 14.5% to 16.4% of couples suffer from infertility, mostly secondary representing 52% [6, 7]. The prevalence of infertility in Ghana is 11.8% [6]. Wide range of factors have been linked to infertility [2, 8, 9], but tubal and uterine abnormalities are the most common causes of female infertility [6, 9–11]. Identified factors may be social, physical, and physiological, and these include but not limited to obesity, abuse of alcohol and tobacco, infections, history of either sexually transmitted diseases (STDs), or pregnancy complications, while orders are unexplained [2, 7–9, 12, 13].

Over the decades, the world has innovated lots of simple but effective diagnostic tools and techniques to identify the potential causes of infertility among couples. Ultrasonography (USG), computed tomography (CT), magnetic resonance imaging (MRI), hysteroscopy, laparoscopy, and hysterosalpingography (HSG) are widely used [14]. Hysterosalpingography is often the first investigation employed in female infertility. The technique uses a contrast medium to visualize the uterine cavity and fallopian tubes, hence able to infer tubal and uterine pathologies [2, 5, 15, 16].

Previous studies on HSG diagnostic outcome reported varied findings [4, 10, 11, 13, 17–22]. About 38% of Nigerian women subpopulation had tubal and uterine abnormalities, and these included tubal blockage (11.38%), uterine fibroid (5.52%), multiple myomata (1.72%), bilateral tubal adhesions (2.07%), uterine adhesions (8.28%), cervical adhesions (2.07%), cervicouterine adhesion (2.41%), hydrosalpinx with spillage (3.45%), and rotated uterus (0.69%) [17].

Another study in Nigeria revealed that 75% with tubal and uterine abnormalities, tubal occlusions (bilateral (14.0%), right (6.0%), and left (7.0%)), uterine fibroids (17.0%), and pelvic adhesion (12.0%) were the most common diagnosis [11]. A study in Iran also reported tubal abnormalities in women with primary and secondary infertility: hydrosalpinx (2.6% and 2.8%, respectively), tubal occlusion (4.7% and 9.8%, respectively), and limited passage of contrast media (2.8% and 3.6%, respectively) [23]. The only study in Ghana found 60.03% of women with various abnormalities, which includes tubal blockage (43.7%) (bilateral (20.5%), left (12.5%), and right (10.6%)) and fibroids (25.4%) [20].

The limited studies on HSG in Ghana indicate an urgent need to improve on data nationwide. This study assessed the spectrum of findings on hysterosalpingography (HSG) in women with infertility in the Central Region and proposed a method for estimating the size of the uterine cavity. The study will provide preliminary data to inform the practice of clinicians, radiologists, and other medical practitioners; add to knowledge of infertility among women in Ghana; and inform further research on infertility in women in the country.

## 2. Materials and Methods

### 2.1. Study Design

This cross-sectional study was conducted at the Department of Imaging of the Cape Coast Teaching Hospital within 8 months period (April to November 2017). Data were collected using semistructured questionnaires. HSG was performed by two radiologists.

### 2.2. Sample Size

A total of 300 women were estimated to attend the Department of Medical Imaging within the 8-month period. Sample size calculation was done using RAOSOFT sample size calculator, assuming minimum sample size for the study taking into consideration the holidays. We estimated 207 patients after including 5% nonrespondent rate.

### 2.3. Sampling Technique

We employed a consecutive sampling to enroll women attending the Department of Medical Imaging.

#### 2.3.1. Inclusion Criteria

The inclusion criteria were as follows:

  Diagnosis of infertility

#### 2.3.2. Exclusion Criteria

The exclusion criteria were as follows:

1. Women with acute infection of the vagina or cervix
2. Women with active vaginal bleeding
3. Pregnant women

### 2.4. Data Collection Procedure

HSG was performed in the preovulatory phase of the menstrual cycle as an outpatient procedure between the 5 and 10^th^ day of the menstrual cycle. The suppository diclofenac (100 mg) was administered 30 minutes prior to the procedure. The patients were placed in the lithotomy position and cleaned, and vaginal speculum was passed to visualize the cervix. A cervical cannula was inserted after air bubbles have been expelled. Approximately 10–20 ml of a water-soluble contrast medium (iopamirol) was injected manually through the cannula under fluoroscopic guidance. Two supine hysterograms were taken. The first image was obtained during the early filling of the uterus and used to evaluate any filling defects or uterine contour abnormality. The second image was taken with the uterus and fallopian tubes fully distended and free intraperitoneal spillage of contrast material seen. An oblique hysterogram was taken in uncertain cases, and no delayed images were taken.

#### 2.4.1. Data Analysis and Interpretation of HSG

Data were entered and analyzed using Statistical Package for Social Sciences (IBM SPSS version 21). We described the data in frequencies, proportions, and mean with standard deviation. Test of statistical association was done using the chi-square test and one-way analysis of variance (ANOVA). All statistical associations were considered significant at *p* value ≤ 0.05. HSG is considered normal when both tubes were well outlined by the free flow of contrast medium, without loculation in the peritoneal cavity and normal uterine outline. HSG was considered abnormal when there was evidence of either unilateral or bilateral tubal obstruction or uterine cavity abnormality.

### 2.5. Ethical Considerations

Ethical approval for this study was obtained from the Ethical Review Committee of the Cape Coast Teaching Hospital. Written informed consent was obtained after the nature of the study was adequately explained to the clients. Clients were assured of data security and confidentiality. They were also informed about the benefit of the research findings and how it will influence the way the procedure is done locally and internationally. Participation was purely voluntary, and they had the option to terminate after been enrolled in the study.

## 3. Results

### 3.1. Demographic and Clinical Presentation of Women

A total of 203 women aged between 19 and 47 years and the mean age of 32.9 years with history of infertility were enrolled. Most of the women were within 30–39 years (61.19%). Secondary infertility (50.74%) was the most common form of infertility, and 58.62% of them have no history of live birth. The commonest duration of infertility (56.16%) was 1 to 4 years. Based on the association between age, duration of infertility, findings on HSG, and indication of HSG, women's age (*p*=0.004) and duration of infertility (*p*=0.034) were found to be significantly associated with the indication of HSG (Table 1).

### 3.2. Findings on Hysterosalpingography

**Figure 1:**
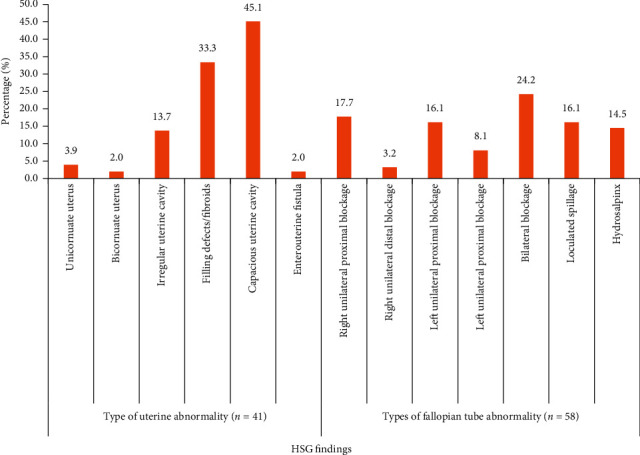
Hysterosalpingography findings in infertile women.

Out of the 203 women studied, eighty-five (41.29%) of them were diagnosed with at least one abnormality on HSG (Table 1). Forty-four (51.3%) of the women had only fallopian tube abnormalities. The common tubal abnormalities were bilateral blockage (24.2%), right unilateral proximal blockage (17.7%), loculated spillage (16.1%), and left unilateral proximal blockage (16.1%) (Figure 1). As shown in Figure 1, 27 (31.8%) women were diagnosed with only uterine abnormality. Capacious uterine cavity (45.1%) and filling defects/fibroids (33.3%) were the common uterine abnormalities. Fourteen (16.5%) women had both fallopian tube and uterine abnormalities.

**Figure 2:**
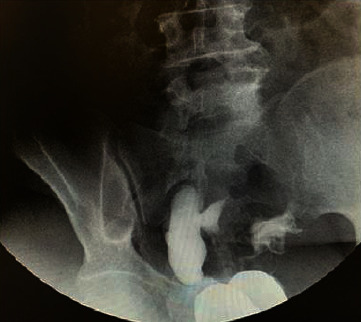
HSG image of right hydrosalpinx and left tubal patency.

On tubal abnormalities, hydrosalpinx and left tubal patency are reported in Figure 2.

**Figure 3:**
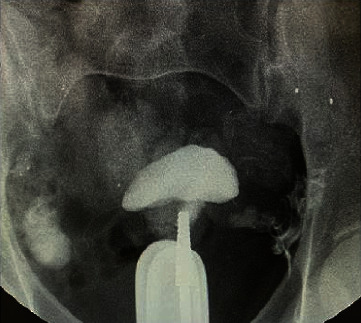
HSG image of bilateral tubal patency and capacious uterine cavity.

We also reported HSG image to demonstrate the bilateral tubal patency and capacious uterine cavity in a participant (Figure 3).

### 3.3. Complimentary Ultrasonography Findings

Forty-nine women with abnormality on HSG had complimentary ultrasound examination, and 51.1% showed abnormalities such as fibroids (78.3%), adenomyosis (4.3%), and 17.4% hydrosalpinx.

### 3.4. Uterine Cavity Size as a Ratio (Jimah Ratio) to the Corno-Iliac Diameter

**Figure 4:**
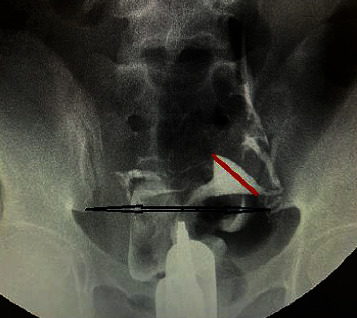
HSG image of Jimah ratio (uterine ratio) = intercornual diameter (red line)/interiliac diameter (black line).

Intercornual diameter (the diameter between the cornua of the uterus) and interiliac diameter (the diameter between lower ends of the right and left iliac bones at the level of the sacroiliac joint) were measured, and ratio (Jimah ratio) between intercornual diameter to interiliac diameter was determined. The HSG measurement of the normal uterine cavity size as a ratio (Jimah ratio) of the corno-iliac diameter is illustrated in Figure 4.

There was significant difference in the mean of intercornual diameter (*p* < 0.001), interiliac diameter (*p* < 0.001), and intercornual diameter to interiliac diameter ratio (*p* < 0.001) for all patients with normal uterine cavities (Table 2).

## 4. Discussion

Hysterosalpingography (HSG) is a common radiographic procedure used in the investigation of female infertility [14]. It is efficient in the detection of fallopian tube and uterine cavity pathologies including tubal occlusion and congenital uterine anomalies [2, 5, 15, 16].

In this study, two hundred and three (203) women with infertility were enrolled, and some had complimentary ultrasound scan (USG). Most of the women presented with secondary infertility (50.74%) which is similar to the findings reported by previous studies in Nigeria [8, 17, 18, 24] and Ghana [20]. On the contrary, studies from different continents reported the predominance of primary infertility in their study subjects: China [25], Iran [23], and India [26]. Our findings are consistent with the assertion that there is a higher prevalence of secondary infertility in women in developing countries compared to primary infertility in developed countries [5, 7, 20]. Previous findings also suggest that the prevalence of risk factors such as sexually transmitted infections, poor reproductive health behavior, iatrogenic health care infections, and medical neglect of precursor conditions of secondary infertility is higher in developing countries [4, 8, 16, 20].

A greater proportion of the women presented with 1 to 4 years duration of infertility, comparable to 4 years as reported by Heis et al. [10], but less than the mean years (6.7 years) as reported by Al-Jaroudi et al. [13]. In this study, age of women (*p*=0.004) and duration of infertility (*p*=0.034) were found to be in association with the forms of infertility. Our findings could not assign any possible reasons for this association. However, aging is likely to influence the occurrence of secondary infertility likely due to the prolonged exposure to the risk factors of infertility.

As seen in this study, eighty-five (41.87%) of the women were diagnosed with at least one or more abnormalities. This is comparable to a study in Switzerland [21] and Nigeria [17] which reported 44% and 37.6%, respectively. Our findings at the Cape Coast Teaching Hospital, Ghana, vary from those of Botwe et al. from the Korle-Bu Teaching Hospital of Ghana, who found that 60.03% of their women had abnormalities on HSG [20]. Many other studies have reported much higher proportions: 98.19% in China [25], 92% in India [26], and 85.8% and 75% in Nigeria [11, 27]. The possible explanation to the disparity in findings is likely due to exposure to infections such as sexually transmitted disease (STD) and pelvic inflammatory disease (PID).

A significant proportion (71.6%) of women who had HSG abnormalities were diagnosed with tubal pathologies. Similar proportions were reported by Aduayi et al. (66.4%) in Nigeria [18], Bhatt et al. (68.4%) in India [26], and Hong et al. (80%) in China [25]. On the contrary, our proportion is much higher than that reported by Niknezhady et al. (11.9%) in Iran [23], Eze et al. (21%) in Nigeria [11], and Schankath et al. (54.7%) in Switzerland [21]. The discrepancies between our finding and previous studies may be due to cultural disparities which predefine the exposure to premarital and extramarital sex and its sequelae such as sexually transmitted diseases and PID [16, 17, 24].

The common tubal abnormalities found were tubal blockage which included bilateral blockage (24.2%) and right unilateral proximal blockage (17.7%), and 16.1% each had loculated spillage and left unilateral proximal blockage. A similar observation was made by Lawan et al. in Nigeria [24].

On HSG, hydrosalpinx is seen as a dilated convoluted tubular structure [27]. The fallopian tube inflammation is aggravated by STDs such as chlamydial or *tuberculosis* of the genital tract [24, 28]. Our proportion for hydrosalpinx (14.5%) among women diagnosed with tubal abnormalities was consistent with previous studies in Ghana [20] and Nigeria [23]. These proportions though low are a major reproductive health problem in women as PID remains a burden to developing countries [4, 8, 16, 17, 24].

Uterine factors remain the second leading cause of infertility among women [5–7, 11, 14, 17, 21, 23]. Our study indicates that almost 50% of women with HSG abnormalities had various forms of uterine pathologies. Individual studies have reported different proportions of uterine abnormalities in Nigeria (33.4% [17] and 56.10%) [27], Switzerland (34.9%) [21], and India (56.4%) [26].

Most of the uterine anomalies consist of capacious uterine cavity (45.1%) and fibroids (33.3%). A similar study in Ghana [20] reported slightly lower proportion (25.4%) of uterine fibroids. On the contrary, much lower proportions were reported by Eze et al. (17.0%) and Abubakar et al. (5.5%) in Nigeria [11, 17]. Complimentary ultrasound (USG) was done for 49 women with uterine abnormality on HSG, and it was observed that 51% had abnormalities such as uterine fibroids (78.3%)

In this study, the mean of intercornual diameter, interiliac diameter (the diameter between lower ends of the right and left iliac bones), and ratio of intercornual diameter to interiliac diameter was taken. This was done for all those with normal uterine findings on HSG, irrespective of the fallopian tube disease. Our findings showed that there was statistically significant difference in the mean of intercornual diameter (*p* < 0.001), intercornual diameter (*p* < 0.001), and the mean ratio of intercornual diameter to interiliac diameter (*p* < 0.001) between those with normal HSG findings and those with only tubal pathologies. The range of intercornual diameter to interiliac diameter ratio (otherwise known as Jimah ratio) is 0.2–0.45, for all women with a mean of 0.36 irrespective of whether there is a fallopian tube abnormality or not. The uterine cavity beyond the upper limit of normal will require further imaging to determine the cause of the enlargement.

Common limitation seen in this study is small sample size. There are five teaching hospitals and other private hospitals in Ghana with imaging departments that provide hysterosalpingography and ultrasonography services; however, this study was conducted in only one of these hospitals (Cape Coast Teaching Hospital). Therefore, the study findings could not be generalized to the entire country. However, this study has provided preliminary data to inform medical practice in our local setting and adds to the knowledge of infertility among women in Ghana.

## 5. Conclusion

Hysterosalpingography is an acceptable imaging modality for the investigation of the uterine cavity and fallopian tubes in infertile women. Secondary infertility was the commonest indication for HSG in the study. Our study indicated that fallopian tube abnormalities are the most common abnormal findings, while capacious uterine cavity, uterine fibroid, and bilateral tubal blockage were the top three abnormalities. The normal uterine cavity size as a ratio (Jimah ratio) of the corno-iliac diameter is 0.2–0.45 with a mean of 0.36 is documented to serve as a local reference value and adds to the existing literature. We recommend further imaging research involving major referral hospitals in Ghana to better assess the spectrum of HSG abnormalities in both normal and infertile women in the country.

## Figures and Tables

**Table 1:** Association between age, duration of infertility, findings on HSG, and type of infertility.

Variable	Indication						Statistical indices	
	Primary		Secondary		Total		*X* ^2^ (df)	*p* value
	*N* = 98	100%	*N* = 103	100%	*N* = 201	100%
*Age*							10.86 (2)	0.004
<30	36	36.73	22	21.36	58	28.86		
30–39	58	59.18	65	63.11	123	61.19		
40+	4	4.08	16	15.53	20	9.95		
*Duration of infertility*							8.67 (3)	0.034
<1	2	2.04	5	4.85	7	3.48		
1_4	62	63.27	52	50.49	114	56.72		
5_10	33	33.67	37	35.92	70	34.83		
>10	1	1.02	9	8.74	10	4.98		
*Findings on HSG*							0.18 (1)	0.674
Normal	59	60.2	59	57.28	118	58.71		
Abnormal	39	39.8	44	42.72	83	41.29		

HSG, hysterosalpingography; *X*^2^, chi-square test d*f*-degree of freedom; significant level at *p* ≤ 0.05.

**Table 2:** Mean and standard deviation of intercornual diameter, interiliac diameter, and ratio of intercornual diameter to interiliac diameter.

HSG findings	Intercornual diameter (*n* = 186)		Interiliac diameter (*n* = 186)		Intercornual to interiliac ratio (Jimah ratio) (*n* = 99)	
	Mean	SD	Mean	SD	Mean	SD
Normal	29.13	4.68	80.65	10.37	0.36	0.06
Abnormal HSG without uterine abnormality	30.33	7.14	80.73	6.64	0.36	0.12
ANOVA	<0.001		<0.001		<0.001	

HSG, hysterosalpingography; SD, standard deviation; ANOVA, one-way analysis of variance; significant level at *p* ≤ 0.05.

## Data Availability

The data used to support the findings of this study are included within the article.
